# False negative and false positive rates in common bile duct brushing cytology, a single center experience 

**Published:** 2018

**Authors:** Bita Geramizadeh, Maryam Moughali, Atefeh Shahim-Aein, Soghra Memari, Ziba Ghetmiri, Alireza Taghavi, Kamran Bagheri Lankarani

**Affiliations:** 1 *Department of Pathology, Medical School, Shiraz University of Medical Sciences, Shiraz, Iran*; 2 *Transplant Research Center, Shiraz University of Medical Sciences, Shiraz, Iran*; 3 *Department of Internal Medicine, Division of Gastroenterology, Shiraz, Iran*; 4 *Health Policy Research Center, Shiraz, Iran *

**Keywords:** Brush cytology, Common bile duct

## Abstract

**Aim:**

In this study we tried to find out the accuracy of biliary tract brushing cytology in our center as the largest referral center in the south of Iran.

**Background:**

Common bile duct brushing cytology has been introduced as the method of choice for the diagnosis of pancreaticobiliary malignancies. However, there have been controversial reports about the sensitivity, specificity and overall accuracy of this method in the English literature.

**Methods:**

During the study period (2012-2016) there has been 166 cases of common bile duct brushing cytology taken during endoscopic retrograde cholangiopancreatography (ERCP). One case has been excluded because of inadequate number of cells in the cytology smear. All the smears have been stained by routine cytologic stains and screened by cytotechnologists and diagnosed by expert cytopathologist. Final diagnosis by biopsy has been considered as the gold standard.

**Results:**

According to the final histologic diagnosis as the gold standard, there were 22 false negative and 7 false positive cases. All of the false positive cases have been suspected cases in the background of primary sclerosing cholangitis. The most common final diagnosis of false negative cytologic diagnoses has been intrahepatic cholangiocarcinoma in which no malignant cell has been identified in the presence of adequate number of normal ductal epithelial cells.

**Conclusion:**

Common bile duct brushing cytology is the method of choice for the diagnosis of pancreaticobiliary tract malignancies; however, having high specificity (90%), the sensitivity is low (56%). Cytologic diagnosis of biliary tract malignancies should be made with caution in the patients with primary sclerosing cholangitis. Also it is important to know that high false negative rate is present in common bile duct brushing cytology especially in the cases of intrahepatic cholangiocarcinoma without extension into extrahepatic ducts.

## Introduction

 Common bile duct (CBD) brushing cytology is an important diagnostic method for the evaluation of pancreatic and biliary tracts abnormalities (1). These abnormalities can be mostly caused by neoplastic or inflammatory processes (2).

CBD brushing procedure was first introduced in 1975 and so far no serious complication has been reported except for mild cholangitis and pancreatitis. However, having high specificity, the sensitivity of the test is not satisfactory (3,4). 

The treatment of pancreaticobiliary abnormalities is composed of different modalities such as installing stents, Whipple’s operation, neoadjuvant therapy and resection or palliative chemotherapy with no surgery. It is very important to have preoperative diagnosis for decision and selection of the type of treatment modality. Brushing cytology is also very important because tissue biopsy is very difficult in this area especially in the presence of CBD stricture and narrowing (1-3).

There are controversial reports regarding this low sensitivity and the diagnostic accuracy of CBD brushing, from different parts of the world. Some of the studies have considered CBD brushing as the method of choice for the diagnosis of biliary tract strictures (5,6). Therefore, in this study we have tried to evaluate the false positive and false negative rate (sensitivity and specificity) of this procedure with the emphasis on the causes of the false positive and negative diagnosis in these cases. 

## Methods

During the study period (2012-2016), we collected all of the cytology smears of CBD and pancreatic duct which have been taken during endoscopic retrograde cholangiopancreatography (ERCP) in 166 cases (Olympus TJF-Q180V). All of the cases were masses, lesions or strictures of the pancreaticobiliary tract and sampling has been performed for the diagnosis of malignancy.

During ERCP, the brush was used to sample the visible lesion, then brushing cytology specimens from ERCP were immediately smeared on the glass slides by the cytotechnologists and then after referral to the cytology lab were stained with Papanicoloau and Wright stains. All of the cases have been screened by the cytotechnologists and then confirmed by the cytopathologist. The cases with at least 5 cellular groups (each containing at least 5 cells) were considered satisfactory; however, presence of any evidence of malignancy or cellular atypia in the smears were considered as satisfactory, no matter how many cells were detected in the cytology smears.

All of the patients were evaluated for the final confirmation by tissue diagnosis of malignancy versus benignancy (as the gold standard). The tissue has been biopsy and/or surgical specimen. All the slides from the tissue and brushing cytology have been seen in blind manner, i.e. neither the cytopathologist (B.G) nor the cytotechnologists (A.S, S.M and Z.G) knew anything about the case.

The results were recorded and then the third person (M.M) evaluated and compared the results to analyze the findings. 

## Results

During the study period 166 cases of brushing cytology of CBD have been received in the cytology laboratory. There was 1 case with inadequate number of cells and very low cellular smears, which has been excluded from the study. 

 In these 165 cases, there were 111 males (67.3%) and 54 female patients (32.7%). and aged between 16 to 91 years (56.44 ±15.95). 

Among these 165 cases, cytology has been reported negative in 130 cases (78.8%) and positive in 35 patients (21.2%), suggestive or suspicious for malignancy.

In 115 cases there were 7 smears with the diagnosis of “atypical cells are present” or “dysplastic cells are seen” which have been considered as positive cytology smear. Final diagnosis, based on the gold standard and tissue biopsy showed 115 benign cases and 50 malignant cases. Comparison of cytologic report with final diagnosis showed 28 true positive and 108 true negative cases. There were 7 false positive cases and 22 false negative cases. According to final diagnosis, the sensitivity of CBD brushing cytology was 56% and specificity was 94%. Among the above mentioned 58 malignant cases, 49 cases have been cholangiocarcinoma from different foci of biliary tract, and 9 cases have been brushing cytology of CBD in pancreatic ductal adenocarcinoma. Eight cases of pancreatic ductal adenocarcinoma have been correctly diagnosed by brushing cytology of CBD, and 1 other case has been falsely negative. There was no false positive cytology in pancreatic ductal adenocarcinoma. Table-1, 2 shows summary of false positive and false negative cases.

**Table 1:** shows cases which have been falsely diagnosed as negative for malignancy by cytology but final diagnosis by tissue as gold standard has been positive for malignancy either originated from the pancreas or biliary tract

Number	Cytology diagnosis by brushing	Final Diagnosis by tissue as gold standard
1	Atypical cell is seen	Cholangiocarcinoma involving CBD* and GB**
2	Atypical cell is seen	Cholangiocarcinoma of CBD
3	Atypical cell is seen	Cholangiocarcinoma of CBD
4	Negative	Cholangiocarcinoma of CBD
5	Negative	Cholangiocarcinoma of CBD
6	Negative	Cholangiocarcinoma of CBD
7	Negative	Cholangiocarcinoma of CBD
8	Negative	Cholangiocarcinoma of CBD
9	Negative	Cholangiocarcinoma of CBD
10	Negative	Intrahepatic and CBD Cholangiocarcinoma
11	Negative	Intrahepatic and CBD Cholangiocarcinoma
12	Negative	Intrahepatic Cholangiocarcinoma
13	Negative	Intrahepatic Cholangiocarcinoma
14	Negative	Intrahepatic Cholangiocarcinoma
15	Negative	Intrahepatic Cholangiocarcinoma
16	Negative	Intrahepatic Cholangiocarcinoma
17	Negative	Intrahepatic Cholangiocarcinoma
18	Negative	Intrahepatic Cholangiocarcinoma
19	Negative	Intrahepatic Cholangiocarcinoma
20	Negative	Intrahepatic Cholangiocarcinoma
21	Negative	Intrahepatic Cholangiocarcinoma
22	Negative	Pancreatic ductal adenocarcinoma

* CBD: Common Bile Duct;

** GB: Gall Bladder

**Table 2:** shows cases with falsely diagnosed as malignant by cytology which have been confirmed by tissue diagnosis as negative for malignancy and no mass or any malignant lesion was detected

Number	Cytologic diagnosis by brushing	Final Diagnosis by tissue as gold standard
1	Suggestive for malignancy	Primary Sclerosing cholangitis
2	Suspicious for malignancy	Primary Sclerosing cholangitis
3	Atypical cells are seen	Primary Sclerosing cholangitis
4	Atypical cells are seen	Primary Sclerosing cholangitis
5	Atypical cells are seen	Primary Sclerosing cholangitis
6	Atypical cells are seen	Primary Sclerosing cholangitis
7	Dysplastic cells are seen	Primary Sclerosing cholangitis

**Figure 1:**
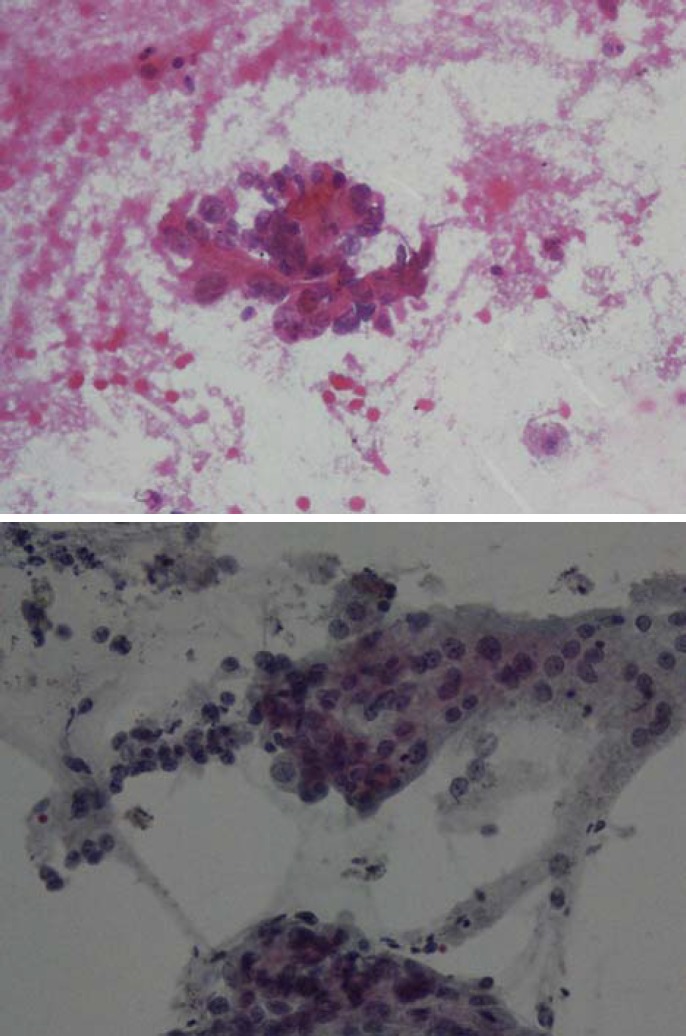
a, b: Smears from a true malignant case show highly atypical cells with irregular chromatin clumping, prominent nucleoli and high N/C ration. (Pap smearX250).

## Discussion

Brushing cytology of the biliary tract has been introduced as the method of choice for the diagnosis of pancreaticobiliary tract lesions (6). The most important diagnostic criteria are the presence of hypercellular smear with overlapped nuclei, with no honey combing appearance containing cells with high N/C ratio, hyperchromasia, irregular chromatin clumping and prominent eosinophilic nucleoli (7) (Fig-1a, b). In the meantime, cytology of biliary tract should be interpreted by an experienced cytopathologist not to miss subtle malignant changes in well differentiated carcinomas. Communication between the cytopathologist and the clinician is also very important for accurate final decision and diagnosis of biliary tract crushing cytology smears (8). 

 Among 165 cases, there were 7 cases with false positive cytologic diagnosis, which have been known cases of primary sclerosing cholangitis (PSC) who has undergone brushing cytology to exclude cholangiocarcinoma on the background of PSC. However, in the patients with PSC there are marked periductal inflammation, fibrosis and epithelial degenerative changes which can be the cause of degenerative atypical changes mimicking malignant process. Figure-1 shows epithelial atypical changes which has been interpreted as epithelial dysplastic changes in a patient with PSC. There are atypical degenerative changes in the presences of many acute inflammatory cells. There have been few studies in the literature emphasizing the high false positive rate of biliary tract brushing cytology in the patients with PSC (9). All of our 7 false positive cases have been reported as either atypical cells or suspicious for malignancy. All of our false positive cases have been reported for the patients with underlying PSC (Table 2).

**Figure 2:**
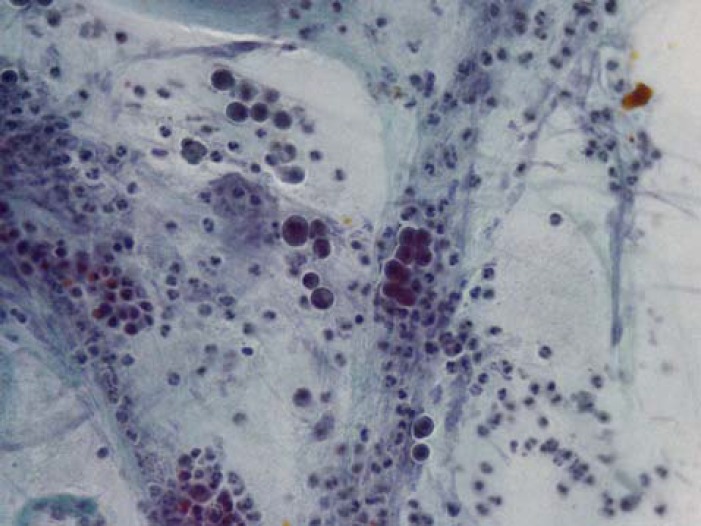
Smears from a false positive case show cellular atypia in the presence of many acute inflammatory cells in the background which have been reported as suspicious for malignancy in cytology report but final diagnosis has been PSC with no malignancy (Pap smear X250)

The most important shortcoming of CBD brushing cytology is high false negativity. In this study we had 22 false negative cases, and as the table-1 shows many of false negative cases have been intrahepatic cholangiocarcinoma. In these cases, despite of good cellularity and adequate number of columnar epithelial cells (Fig-2), there have been no malignant cells in all of the smears; therefore, it seems that clinicians should be cautious about a negative brushing cytology of CBD in the suspected cases of intrahepatic cholangiocarcinoma. Some studies have recommended a combination of brush cytology and forceps biopsy to improve the diagnostic yield (11). Some recent studies have used long cytobrushes to brush larger and longer areas of the biliary tract to overcome this shortcoming of low cellularity to decrease false negative rates (12-17).
